# Treatment patterns for patients initiating novel acute migraine specific medications (nAMSMs) in the context of monoclonal antibodies (mAbs) targeting the calcitonin gene-related peptide (CGRP) pathway

**DOI:** 10.1186/s10194-023-01678-y

**Published:** 2023-11-09

**Authors:** Zifan Zhou, Robert Urman, Karminder Gill, Andrew S. Park, Fiston Vuvu, Leah B. Patel, Jingsong Lu, Rolin L. Wade, Lindsay Frerichs, Mark E. Bensink

**Affiliations:** 1 IQVIA, Falls Church, VA USA; 2 Amgen Inc., Thousand Oaks, CA USA; 3 Washington University School of Medicine, St. Louis, MO USA; 4 Benofit Consulting, Brisbane, QLD Australia

**Keywords:** Acute migraine therapy, Prophylactic/preventive treatment, CGRP, Migraine

## Abstract

**Background:**

New acute and preventive migraine medications are available, but data on current treatment patterns are limited. This study describes migraine treatment patterns among patients initiating novel acute migraine specific medications (nAMSMs), overall and by prior use of anti-calcitonin gene-related peptide (CGRP) pathway monoclonal antibodies (mAbs).

**Methods:**

In this retrospective cohort study using IQVIA open-source pharmacy and medical claims data, we identified patients with ≥ 1 claim for a nAMSM (ubrogepant, rimegepant, lasmiditan) between 01/01/2020 and 09/30/2020 (index period). Patients were indexed on their first nAMSM claim and stratified into 2 cohorts: patients with prior mAb use (≥ 1 claim for erenumab, fremanezumab, galcanezumab in the 6-month pre-index period) or patients without prior mAb use. Treatment patterns were assessed during the 6-month post-index period.

**Results:**

Overall, 78,574 patients were identified (63% indexed on ubrogepant, 34% on rimegepant, and 3% on lasmiditan) with 26,656 patients (34%) having had prior mAb use. In the pre-index period, 79% of patients used non-mAb preventive medications and 75% of patients used acute medications. Following the index nAMSM claim, 65% of patients had ≥ 1 refill and 21% had ≥ 4 refills of their index nAMSM; 10% of patients switched to another nAMSM. Post-index mAb use was observed in 82% of patients with a prior mAb and 15% of patients without. Among patients with pre- and post-index use of acute medications, 38% discontinued ≥ 1 acute medication class in the post-index period. Among patients with concomitant use of traditional preventive medications at index, 30% discontinued ≥ 1 concomitant preventive anti-migraine medication in the post-index period.

**Conclusions:**

Most patients initiating nAMSMs had prior treatment with acute and preventive medications. Approximately one-third of patients had prior treatment with anti-CGRP pathway mAbs. After starting nAMSMs, more than one-third of patients discontinued at least one traditional acute medication and one-third of patients discontinued at least one traditional preventive medication.

Despite nAMSM initiation, most patients with prior anti-CGRP pathway mAb use continued mAb use. Around 15% of patients without a prior mAb newly started a mAb. These results provide insight into how nAMSMs and mAbs have been integrated into clinical management of migraine in the real-world.

**Supplementary Information:**

The online version contains supplementary material available at 10.1186/s10194-023-01678-y.

## Background

Migraine is one of the most prevalent neurological diseases worldwide [1]. It is characterized by moderate to severe headache that is often described as pounding or pulsing and may be accompanied by symptoms such as nausea, vomiting, and sensitivity to light and sounds. Estimates have shown that migraine affected about 16% of the population aged 12 and older in the United States (US) in 2020 [2]. Migraine poses a large and increasing burden on patients and society, accounting for roughly 4 million emergency department visits and over 4.3 million office visits in 2016 [2].

Calcitonin gene-related peptide (CGRP) plays a crucial role in migraine pathophysiology and has been established as an important target for both preventive and acute treatments [3, 4]. Monoclonal antibodies (mAbs) that target the CGRP pathway, including erenumab-aooe, galcanezumab-gnlm, fremanezumab-vfrm and eptinezumab-jjmr, are a class of prophylactic treatment that were first introduced to the U.S. market in 2018. Novel acute migraine specific medications (nAMSMs) have also emerged in recent years, including small molecule CGRP receptor antagonists, known collectively as gepants, as well as a new first in class serotonin 5-HT1F receptor agonist (lasmiditan) [5]. Gepants, which includes rimegepant (approved in February 2020) and ubrogepant (approved in December 2019), and lasmiditan (approved in October 2019), offer treatment options for acute migraine attacks when traditional therapies are not effective or are contraindicated [6].

Although prior studies have evaluated real-world treatment patterns for patients initiating mAbs targeting the CGRP pathway [7–15], there are limited real-world data on the use of nAMSMs in the context of anti-CGRP pathway mAbs. The safety and tolerability of nAMSMs in combination with mAbs has been explored in the literature. A phase 1b study (*n* = 40) investigating the pharmacokinetic (PK) profile and safety of ubrogepant with galcanezumab or ubrogepant with erenumab, identified no significant changes to the PK profile of ubrogepant and no safety concerns [16]. The combination of ubrogepant and anti-CGRP pathway mAbs has also been reported in a survey-based, real-world study [17]. In that study, 59% (62 of 105) patients with concurrent use of ubrogepant and anti-CGRP pathway mAbs had a response rate (defined as the proportion of patients with headache relief for ≥ 75% of all treated attacks at 2 h) and a safety profile that was similar to patients on anti-CGRP pathway mAbs alone.

The objective of this study was to describe migraine treatment patterns among patients initiating nAMSMs, overall and by prior use of anti-CGRP pathway mAbs.

## Methods

### Study design and data sources

This was a retrospective cohort study using IQVIA open-source US pharmacy (LRx) and medical (Dx) claims data. The LRx database contains more than 250 million unique patients, across multiple payer and coverage structures including cash and manufacturers’ discount and coupon programs [7]. Data are collected via direct feeds from pharmacy suppliers capturing adjudicated and dispensed prescriptions sourced from retail, mail, long-term care and specialty pharmacies [7] including patient demographics, payer type, product information, 3-digit zip as well as prescription relevant information including prescriber, date of service, refill (medication claims after the initial prescription claim) number, quantity dispensed and days supply. The Dx medical claims database provides patient-level diagnoses, procedures, and administered therapeutics for over 1.5 billion claims per year for visits to US office-based physician, ambulatory, and general healthcare sites; and captures claims from commercial, Medicare, Medicaid, and cash payers [7]. Dx data was used in this study to obtain patients’ comorbidity history. All data in both databases are compliant with the Health Insurance Portability and Accountability Act to protect patients’ privacy.

### Patient selection

Patients with ≥ 1 pharmacy claim for an nAMSM (ubrogepant, rimegepant, or lasmiditan) in the LRx database between January 1, 2020, through September 30, 2020 (the index period), were identified and assigned an index date on the date of their first nAMSM claim. This index period was selected to allow for a 6-month pre-index (6-month period prior to the index date) and a 6-month post-index period (6-month period after and including the index date) for assessment of patient characteristics. Additional patient identification criteria required for study inclusion were ≥ 18 years of age on the index date, linkage to Dx, ≥ 1 pharmacy claim of any type prior to the 6-month pre-index period and ≥ 1 pharmacy claim of any type after the end of the 6-month post-index period (ensuring pharmacy claims visibility) in LRx, use of a pharmacy that consistently contributed data during the 6-month pre-index and the 6-month post-index periods in LRx, ≥ 2 medical claims ≥ 30 days apart in the 6-month pre-index period (ensuring medical claims visibility for comorbidities) in Dx, and no data quality issues (defined as having ≥ 2 distinct index medications on index date or missing age or sex information).

### Study variables

#### Demographics and clinical characteristics

Demographic characteristics (age and sex), prescriber of index nAMSM, and index payer type were assessed on the index date. Comorbidities were identified using International Classification of Diseases (ICD)-10 codes in the pre-index period with the most frequent comorbidities (> 5% of patients) being presented. In addition, the Charlson Comorbidity Index score was computed for each patient based on comorbidities identified during the pre-index period [18, 19].

#### Medications of interest

For nAMSMs (ubrogepant, rimegepant, or lasmiditan), the index claim, dose, and quantity dispensed were identified. In the 6 months following the index nAMSM, the number of refills of the index nAMSM and time between refills were assessed, as well as any switches from the index nAMSM to a different nAMSM.

Use of anti-CGRP pathway mAbs, which included subcutaneously administered erenumab, fremanezumab, and galcanezumab, was evaluated in the pre-index period and in the post-index period. Intravenous administered eptinezumab was excluded from the analysis due to limited sample size.

Use of traditional acute and preventive anti-migraine medications (defined as having ≥ 1 claim) was evaluated in the pre-/post-index periods. Traditional acute anti-migraine medications included triptans, opioids, ergots (dihydroergotamine and ergotamine-containing products), and non-steroidal anti-inflammatory drugs (NSAIDs). Traditional preventive anti-migraine medications included select anticonvulsants (carbamazepine, gabapentin, levetiracetam, pregabalin, topiramate, valproate sodium, valproic acid, divalproex sodium, zonisamide), select antihypertensives (atenolol, bisoprolol, metoprolol, nadolol, nebivolol, pindolol, propranolol, timolol, verapamil, candesartan, clonidine including transdermal patches, lisinopril, olmesartan), select antidepressants (duloxetine, desvenlafaxine, venlafaxine, amitriptyline, desipramine, doxepin, imipramine, nortriptyline, protriptyline, clomipramine, escitalopram, citalopram, sertraline, mirtazapine), select botulinum toxins (abobotulinumtoxin A injection, incobotulinumtoxin A injection, onabotulinumtoxin A injection, rimabotulinumtoxin B injection), and other medications (carisoprodol, cyproheptadine, guanfacine, memantine, methysergide, milnacipran, tizanidine).

Discontinuation of traditional acute and preventive anti-migraine medications was reported at the class level. The discontinuation definitions differed for traditional acute and preventive anti-migraine medications, as traditional acute anti-migraine medications were taken as needed, while traditional preventive anti-migraine medications were taken on a scheduled basis. Discontinuation of traditional acute anti-migraine medications was evaluated among the subset of patients with ≥ 1 claim for the class in the pre-index period and ≥ 1 claim for the class in the post-index period and was defined as no claims for the same class in the 60 days prior to the end of the post-index period. Discontinuation of traditional preventive anti-migraine medications was evaluated among the subset of patients with concomitant preventive medication use on the index date (≥ 1 claim for the preventive medication class with days supply overlapping with the index date) and was defined as ≥ 60-day gap (≥ 90-day gap for botulinum toxins) in days supply of the preventive medication class in the post-index period.

Changes in equivalent dose of traditional acute anti-migraine medications (triptans, opioids, and ergots) from the pre-index period to the post-index period were measured among the subset of patients with pre-index acute anti-migraine medication use who continued use of the traditional acute anti-migraine medication during the post-index period. Triptans were converted to oral sumatriptan milligram (mg) equivalents, opioids were converted to oral morphine mg equivalents, and ergots were converted to oral ergotamine mg equivalents During each period (pre-/post-index period), the patient’s equivalent dose per month was calculated using the following formula:

$$Equivalent\;dose\;per\;month=\frac{Sum\;of\;quantity\;during\;the\;period\;\ast\;dose\;equivalent\;strength}6$$

The change in equivalent dose was calculated as the post-index equivalent dose minus the pre-index equivalent dose, resulting in negative values that represent a reduction in dose and positive values representing an increase in dose in the post-index period.

### Statistical analyses

All analyses were descriptive and were performed using SAS version 9.3 (SAS Institute Inc., Cary, NC). Frequencies and percentages are presented for categorical variables, while means, standard deviations, and medians are presented for continuous variables. Analyses were conducted among the entire study population (all patients initiating nAMSMs) and then stratified among patients with prior mAb use (≥ 1 pre-index claim for erenumab, fremanezumab, or galcanezumab) and patients without prior mAb use.

## Results

**Fig. 1:**
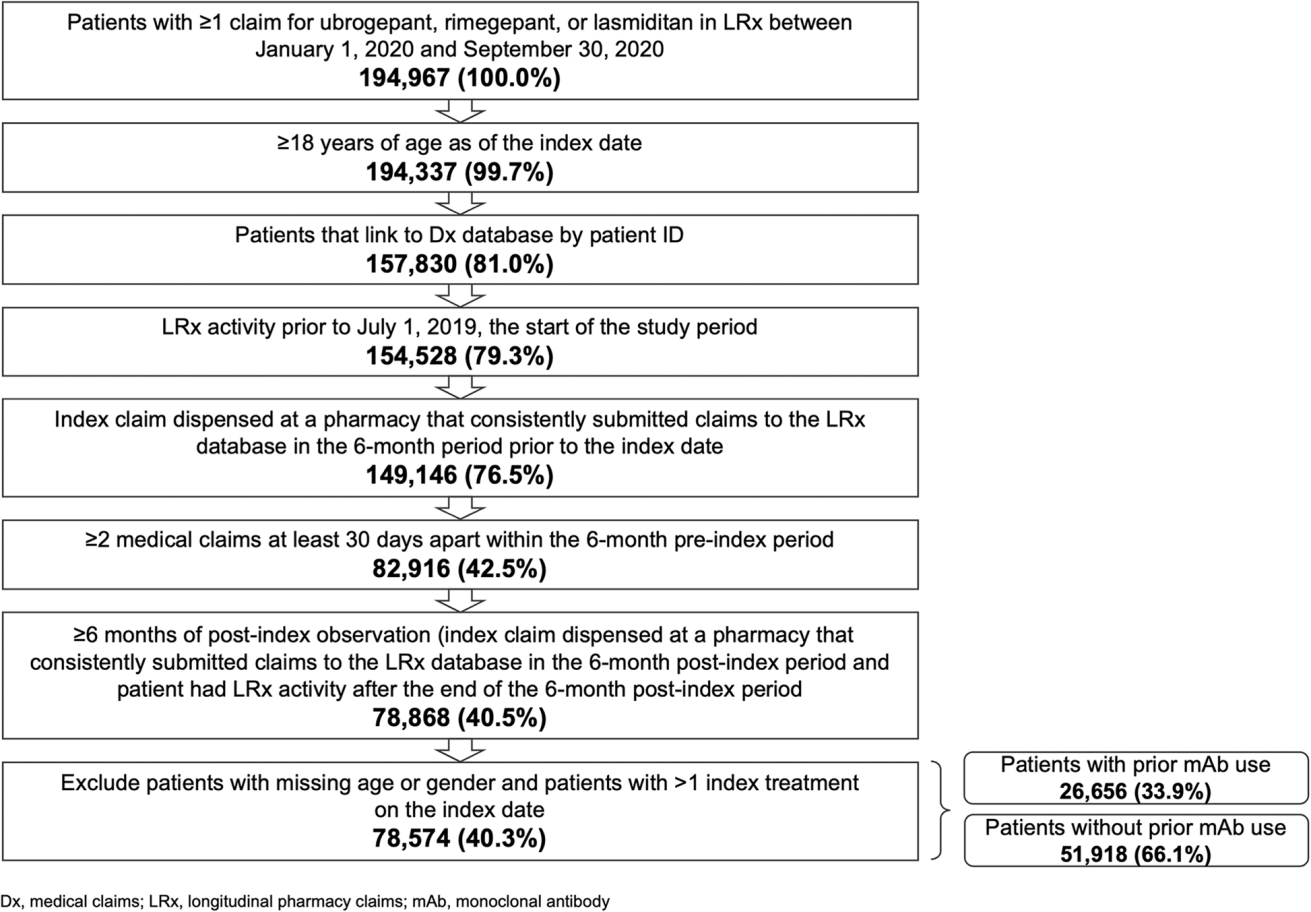
Patient sample selection and attrition

After applying the patient selection criteria (Fig. 1), 78,574 patients were identified for the study sample; 26,656 patients (34%) had prior mAb use and 51,918 (66%) did not have prior mAb use.

### Demographic and baseline clinical characteristics

Demographics and pre-index clinical characteristics are displayed in Table 1. Mean (SD) age was 47 (13) years and 87% were female. Age and sex were similar for patients with and without prior mAb use. Patients had a low burden of comorbidities; 74% of patients had a Charlson Comorbidity index score of 0. The most common comorbidities were anxiety, hypertension, depression, and asthma. The frequency of depression was 16% in patients with prior mAb use and 14% in patients without prior mAb use.

**Table 1:** Demographics and clinical characteristics

	**Overall** ***N* = 78,574**	**Patients with prior mAb use**^**a**^ ***N* = 26,656**	**Patients without prior mAb use**^**a**^ ***n* = 51,918**
**Demographics**			
Age, mean (SD)	46.7 (13.3)	47.0 (12.8)	46.5 (13.5)
Female sex, n (%)	68,631 (87.4%)	23,319 (87.5%)	45,312 (87.3%)
**Index payer type, n (%)**			
Commercial	50,607 (64.4%)	17,002 (63.8%)	33,605 (64.7%)
Discounts/coupons	16,118 (20.5%)	5,234 (19.6%)	10,884 (21.0%)
Medicaid	7,973 (10.2%)	2,955 (11.1%)	5,018 (9.7%)
Cash	2,997 (3.8%)	1,134 (4.3%)	1,863 (3.6%)
Medicare	873 (1.1%)	329 (1.2%)	544 (1.1%)
**Prescriber for index medication, n (%)**			
Neurologist	37,375 (47.6%)	13,790 (51.7%)	23,585 (45.4%)
Nurse practitioner/ physician assistant	23,915 (30.4%)	8,553 (32.1%)	15,362 (29.6%)
Primary care physician	11,719 (14.9%)	2,401 (9.0%)	9,318 (18.0%)
Pain specialist	1,194 (1.5%)	479 (1.8%)	715 (1.4%)
Other	4,371 (5.6%)	1,433 (5.4%)	2,938 (5.7%)
**Charlson comorbidity index score, n (%)**			
0	57,871 (73.7%)	19,770 (74.2%)	38,101 (73.4%)
1	12,356 (15.7%)	4,174 (15.7%)	8,182 (15.8%)
2	5,062 (6.4%)	1,665 (6.3%)	3,397 (6.5%)
3 +	3,285 (4.2%)	1,047 (3.9%)	2,238 (4.3%)
**Most frequent comorbidities (> 5% of patients), n (%)**			
Anxiety	14,271 (18.2%)	4,994 (18.7%)	9,277 (17.9%)
Hypertension	13,792 (17.6%)	4,538 (17.0%)	9,254 (17.8%)
Depression	11,429 (14.6%)	4,335 (16.3%)	7.094 (13.7%)
Asthma	5,513 (7.0%)	1,986 (7.5%)	3,527 (6.8%)

*mAb* monoclonal antibody, *SD* standard deviation

^a^ Prior mAb (erenumab, galcanezumab, fremanezumab) use was evaluated during the 6-month period prior to the index date

Index nAMSM were primarily prescribed by neurologist (48%), followed by nurse practitioner/physician assistant (30%), and primary care provider (15%). The index nAMSM was more frequently prescribed by a neurologist for patients with prior mAb use than patients without prior mAb use (52% vs. 45%). Conversely, the index nAMSM was more frequently prescribed by a primary care physician for patients without prior mAb use than patients with prior mAb use (18% vs. 9%). The majority of patients (64%) had commercial insurance and 21% of patients used discounts and coupons for their index nAMSM prescription. Details on payer switching during the post-index period are provided in Supplement Table 1.

### Pre-index traditional acute and preventive anti-migraine medications

In the pre-index period, 75% of patients used traditional acute anti-migraine medications and 79% used traditional preventive anti-migraine medications (Table 2). Patients with prior mAb use had greater pre-index use of traditional acute and preventive anti-migraine medications than patients without prior mAb use (82% vs. 72% for traditional acute anti-migraine medications, 84% vs. 77% for traditional preventive anti-migraine medications).

**Table 2:** Patients with use of traditional acute and preventive migraine medications during the 6-month pre-index period, overall and by prior mAb use

	**Overall** ***N* = 78,574**	**Patients with prior mAb use**^**a**^ ***N* = 26,656**	**Patients without prior mAb use**^**a**^ ***n* = 51,918**
**Acute migraine medication use, n (%)**	**59,290 (75.5%)**	**21,842 (81.9%)**	**37,448 (72.1%)**
Triptans	36,054 (45.9%)	14,182 (53.2%)	21,872 (42.1%)
Opioids	29,692 (37.8%)	11,116 (41.7%)	18,576 (35.8%)
NSAIDs	26,644 (33.9%)	10,118 (38.0%)	16,526 (31.8%)
Ergots	1,255 (1.6%)	718 (2.7%)	537 (1.0%)
**Preventive migraine medication use (n, %)**	**62,428 (79.5%)**	**22,378 (84.0%)**	**40,050 (77.1%)**
Select antidepressant medications^b^	37,753 (48.1%)	14,108 (52.9%)	23,645 (45.5%)
Select anticonvulsant medications^c^	34,930 (44.5%)	13,197 (49.5%)	21,733 (41.9%)
Select antihypertensive medications^d^	24,751 (31.5%)	9,243 (34.7%)	15,508 (29.9%)
Other medications that prevent migraines^e^	12,494 (15.9%)	5,353 (20.1%)	7,141 (13.8%)
Select botulinum toxin medications^f^	9,669 (12.3%)	3,378 (12.7%)	6,291 (12.1%)

*mAb* monoclonal antibody, *NSAIDs* non-steroidal anti-inflammatory drugs

^a^Prior mAb (erenumab, galcanezumab, fremanezumab) use was evaluated during the 6-month period prior to the index date

^b^Select antidepressant medications included oral formulations of duloxetine, desvenlafaxine, venlafaxine, amitriptyline, desipramine, doxepin, imipramine, nortriptyline, protriptyline, clomipramine, escitalopram, citalopram, sertraline, and mirtazapine

^c^Select anticonvulsant medications included oral formulations of carbamazepine, gabapentin, levetiracetam, pregabalin, topiramate, valproate sodium, valproic acid, divalproex sodium, and zonisamide

^d^Select antihypertensive medications included oral formulations (unless noted otherwise) of atenolol, bisoprolol, metoprolol, nadolol, nebivolol, pindolol, propranolol, timolol, verapamil, candesartan, clonidine (oral and transdermal patch formulations), lisinopril, and olmesartan

^e^Other medications that prevent migraines included oral formulations of carisoprodol, cyproheptadine, guanfacine, memantine, methysergide, milnacipran, and tizanidine

^f^Select botulinum toxin medications included abobotulinumtoxinA injection, incobotulinumtoxinA injection, onabotulinumtoxinA injection, and rimabotulinumtoxinB injection. These medications were identified with National Drug Codes (NDCs) on prescription claims and Healthcare Procedural Classification System (HCPCS) codes on medical claims

### nAMSM prescription patterns

In the overall study sample, 27,140 (35%) of patients indexed on rimegepant, 49,187 (63%) indexed on ubrogepant, and 2,247 (3%) indexed on lasmiditan (Table 3). Index treatment with ubrogepant or with lasmiditan was higher among patients with prior mAb use than patients without prior mAb use, while index treatment with rimegepant was lower among patients with prior mAb use. Among patients who indexed on ubrogepant, 67% had an index dose of 50 mg and 33% had an index dose of 100 mg. Among patients who indexed on lasmiditan, 33% had an index dose of 50 mg and 67% had an index dose of 100 mg. The mean (SD) quantity dispensed for the index nAMSM was 10.0 (4.3) and was similar across nAMSMs (ubrogepant 11.1 [4.9], rimegepant 8.2 [1.9] and lasmiditan 8.1 [2.9]; data not shown) as well as for patients with and for patients without prior mAb use.

**Table 3:** Index and post-index nAMSM treatment patterns, overall and by prior mAb use

	**Overall** ***N* = 78,574**	**Patients with prior mAb use**^**a**^ ***N* = 26,656**	**Patients without prior mAb use**^**a**^ ***n* = 51,918**
**Index medication and dose, n (%)**			
**Rimegepant**	**27,140 (34.5%)**	**8,465 (31.8%)**	**18,675 (36.0%)**
Rimegepant 75mg	27,140 (100.0%)	8,465 (100.0%)	18,675 (100.0%)
**Ubrogepant**	**49,187 (62.6%)**	**17,084 (64.1%)**	**32,103 (61.8%)**
Ubrogepant 50mg	33,065 (67.2%)	10,961 (64.2%)	22,104 (68.9%)
Ubrogepant 100mg	16,101 (32.7%)	6,115 (35.8%)	9,986 (31.1%)
**Lasmiditan**	**2,247 (2.9%)**	**1,107 (4.2%)**	**1,140 (2.2%)**
Lasmiditan 50mg	731 (32.5%)	319 (28.8%)	412 (36.1%)
Lasmiditan 100mg	1,513 (67.3%)	788 (71.2%)	725 (63.6%)
**Index nAMSM quantity dispensed**			
Mean (SD) [median]	9.98 (4.28) [10]	9.97 (4.13) [10]	9.98 (4.35) [10]
**Number of refills**^**b**^** for index medication, n (%)**			
0 refills	27,136 (34.5%)	7,698 (28.9%)	19,438 (37.4%)
1 refill	15,152 (19.3%)	4,996 (18.7%)	10,156 (19.6%)
2 refills	11,110 (14.1%)	3,846 (14.4%)	7,264 (14.0%)
3 refills	8,378 (10.7%)	3,141 (11.8%)	5,237 (10.1%)
4 + refills	16,798 (21.4%)	6,975 (26.2%)	9,823 (18.9%)
**Refills**^**b**^** for index therapy among patients with ≥ 1 refill**			
Patients with ≥ 1 refill, n (%)	51,438 (65.5%)	18,958 (71.1%)	32,480 (62.6%)
Number of refills, mean (SD) [median]	2.91 (1.94) [2]	3.11 (2.03) [3]	2.80 (1.88) [2]
Days from index date to first refill, mean (SD) [median]	53.3 (38.3) [39]	50.3 (36.6) [37]	55.0 (39.2) [41]
**Switch from index nAMSM to a different nAMSM**			
Patients with ≥ 1 nAMSM switch	8,122 (10.3%)	4,091 (15.4%)	4,031 (7.8%)

*mAb* monoclonal antibody, *nAMSMs* novel acute migraine specific medications, *SD* standard deviation

^a^Prior mAb (erenumab, galcanezumab, fremanezumab) use was evaluated during the 6-month period prior to the index date

^b^A refill was defined as a claim for the index medication during the post-index period (excluding the index date) unless specified otherwise

Following the index nAMSM claim, 65% of patients had ≥ 1 refill and 21% had ≥ 4 refills of their index nAMSM (Table 3). Patients with prior mAb use more frequently had ≥ 1 refill for their index nAMSM than patients without prior mAb use (71% vs. 63%). The mean (SD) number of index nAMSM medication refills for patients with a refill was 3.1 (2.0) for patients with prior mAb use and 2.8 (1.9) for patients without prior mAb use. The percentage of patients with a switch to a non-index nAMSM was higher in nAMSM patients with prior mAb use than in those without prior mAb use (15% vs. 8%).

### mAb prescription patterns

Among the 34% of patients with prior mAb use, the mAb used in the pre-index period closest to the nAMSM index date was erenumab for 11,402 (43%) patients, galcanezumab for 10,520 (39%) patients, and fremanezumab for 4,734 (18%) patients. Patients with prior mAb use averaged 4.0 (SD 2.0) pre-index mAb claims. One or more post-index mAb claims were observed in 82% of patients with prior mAb use and 15% of patients without prior mAb use. The mean (SD) number of post-index mAb claims was 4.6 (1.9) in patients with prior mAb use and 3.4 (1.9) in patients without prior mAb use (data not shown).

### Change in traditional acute and preventive anti-migraine medications

Post-index discontinuation results for traditional acute and preventive anti-migraine medications are displayed in Table 4. Among the subset of patients with pre-index use of traditional acute migraine medications (*n* = 59,290), 71% (*n* = 42,338) had pre- and post-index use, and 38% of these patients subsequently discontinued ≥ 1 acute anti-migraine medication class post-index; discontinuation was 32% for triptans, 22% for opioids, 45% for ergots, and 39% for NSAIDs. The proportion of patients that discontinued traditional acute anti-migraine medications was 37% among patients with prior mAb use and 38% among patients without prior mAb use. Among patients with concomitant use of traditional preventive anti-migraine medications at index, 30% of patients overall, 27% of patients with prior mAb use, and 32% of patients without prior mAb use discontinued ≥ 1 concomitant preventive anti-migraine medications post-index.

**Table 4:** Discontinuation of traditional acute and preventive migraine medications during the 6-month post-index period, overall and by prior mAb use

	**Overall** ***N* = 78,574**	**Patients with prior mAb use**^**a**^ ***N* = 26,656**	**Patients without prior mAb use**^**a**^ ***N* = 51,918**
**Discontinuation of acute migraine treatments**^**b**^			
Pre-index acute migraine treatment use, n (%)	59,290 (75.5)	21,842 (81.9)	37,448 (72.1)
Patients with ≥ 1 claim for acute migraine medication pre- and post-index, n (%)	42,338 (71.4)	16,826 (77.0)	25,512 (68.1)
Patients with discontinuation of any acute migraine medication class, n (%)^c^	15,953 (37.7)	6,157 (36.6)	9,796 (38.4)
Pre-index triptan use, n (%)	36,054 (45.9)	14,182 (53.2)	21,872 (42.1)
Patients with ≥ 1 claim for triptan pre- and post-index, n (%)	20,677 (57.4)	9,081 (64.0)	11,596 (53.0)
Patients with triptan discontinuation, n (%)^c^	6,665 (32.2)	2,686 (29.6)	3,979 (34.3)
Pre-index opioid use, n (%)	29,692 (37.8)	11,116 (41.7)	18,576 (35.8)
Patients with ≥ 1 claim for opioid pre- and post-index, n (%)	20,745 (69.9)	8,121 (73.1)	12,624 (68.0)
Patients with opioid discontinuation, n (%)^c^	4,610 (22.2)	1,687 (20.8)	2,923 (23.2)
Pre-index ergot use, n (%)	1,255 (1.6)	718 (2.7)	537 (1.0)
Patients with ≥ 1 claim for ergot pre- and post-index, n (%)	501 (39.9)	300 (41.8)	201 (37.4)
Patients with ergot discontinuation, n (%)^c^	223 (44.5)	138 (46.0)	85 (42.3)
Pre-index NSAID use, n (%)	26,644 (33.9)	10,118 (38.0)	16,526 (31.8)
Patients with ≥ 1 claim for NSAID pre- and post-index, n (%)	16,101 (60.4)	6,518 (64.4)	9,583 (58.0)
Patients with NSAID discontinuation, n (%)^c^	6,253 (38.8)	2,396 (36.8)	3,857 (40.3)
**Discontinuation of concomitant preventive migraine medications**^**d,e**^			
Pre-index preventive migraine medication use, n (%)	62,428 (79.5)	22,378 (84.0)	40,050 (77.1)
Patients with concomitant use of ≥ 1 preventive migraine medication, n (%)	55,166 (88.4)	19,671 (87.9)	35,495 (88.6)
Patients with discontinuation of any concomitant preventive medication, n (% of patients with concomitant use)	16,593 (30.1)	5,368 (27.3)	11,225 (31.6)
Pre-index anticonvulsant use, n (%)	34,930 (44.5)	13,197 (49.5)	21,733 (41.9)
Patients with concomitant use of select anticonvulsant medications^f^, n (%)	27,058 (77.5)	10,028 (76.0)	17,030 (78.4)
Patients with discontinuation of concomitant select anticonvulsant medications, n (% of patients with concomitant use)	6,205 (22.9)	1,872 (18.7)	4,333 (25.4)
Pre-index antihypertensive use, n (%)	24,751 (31.5)	9,243 (34.7)	15,508 (29.9)
Patients with concomitant use of select antihypertensive medications^g^, n (%)	19,788 (79.9)	7,277 (78.7)	12,511 (80.7)
Patients with discontinuation of concomitant select antihypertensive medications, n (% of patients with concomitant use)	3,794 (19.2)	1,208 (16.6)	2,586 (20.7)
Pre-index antidepressant use, n (%)	37,753 (48.1)	14,108 (52.9)	23,645 (45.5)
Patients with concomitant use of select antidepressant medications^h^, n (%)	30,283 (80.2)	11,307 (80.1)	18,976 (80.3)
Patients with discontinuation of concomitant select antidepressant medications, n (% of patients with concomitant use)	5,324 (17.6)	1,670 (14.8)	3,654 (19.3)
Pre-index botulinum toxin use, n (%)	9,669 (12.3)	3,378 (12.7)	6,291 (12.1)
Patients with concomitant use of select botulinum toxin medications^i^, n (%)	8,033 (83.1)	2,682 (79.4)	5,351 (85.13)
Patients with discontinuation of concomitant select botulinum toxin medications, n (% of patients with concomitant use)	1,252 (15.6)	397 (14.8)	855 (16.0)
Pre-index other medication that prevent migraine use, n (%)	12,494 (15.9)	5,353 (20.1)	7,141 (13.8)
Patients with concomitant use of other medications that prevent migraines^j^, n (%)	7,680 (61.5)	3,382 (63.2)	4,298 (60.2)
Patients with discontinuation of concomitant other medications that prevent migraines, n (% of patients with concomitant use)	2,354 (30.7)	969 (28.7)	1,385 (32.2)

*mAb* monoclonal antibody, *NSAIDs* non-steroidal anti-inflammatory drugs

^a^Prior mAb (erenumab, galcanezumab, fremanezumab) use was evaluated during the 6-month period prior to the index date

^b^Discontinuation of acute migraine medication was defined as no claims for the same therapeutic class of acute treatment during the 60 days prior to the end of the post-index period

^c^Amongst patients with ≥ 1 claim pre- and post-index

^d^Concomitant preventive anti-migraine therapy with novel acute migraine specific medication (nAMSM) was defined as a claim for ≥ 1 preventive medication class in the pre-index period with days supply that overlaps with the index date

^e^Discontinuation of concomitant preventive treatment was defined as a ≥ 60-day gap (≥ 90 days for botulinum toxins which are administered every 3 months) in preventive medication (after the end of days supply) in the post-index period

^f^Select anticonvulsant medications included oral formulations of carbamazepine, gabapentin, levetiracetam, pregabalin, topiramate, valproate sodium, valproic acid, divalproex sodium, and zonisamide

^g^Select antihypertensive medications included oral formulations (unless noted otherwise) of atenolol, bisoprolol, metoprolol, nadolol, nebivolol, pindolol, propranolol, timolol, verapamil, candesartan, clonidine (oral and transdermal patch formulations), lisinopril, and olmesartan

^h^Select antidepressant medications included oral formulations of duloxetine, desvenlafaxine, venlafaxine, amitriptyline, desipramine, doxepin, imipramine, nortriptyline, protriptyline, clomipramine, escitalopram, citalopram, sertraline, and mirtazapine

^i^Select botulinum toxin medications included abobotulinumtoxinA injection, incobotulinumtoxinA injection, onabotulinumtoxinA injection, and rimabotulinumtoxinB injection. These medications were identified with NDCs on prescription claims and HCPCS on medical claims

^j^Other medications that prevent migraines included oral formulations of carisoprodol, cyproheptadine, guanfacine, memantine, methysergide, milnacipran, and tizanidine

Table 5 shows the changes in equivalent dose of traditional acute anti-migraine medications (triptans, opioids, and ergots) among the patients with continuing use of the traditional acute anti-migraine medications during the post-index period (*n* = 14,012 for triptans, *n* = 16,135 for opioids, and *n* = 278 for ergots). Among continuing triptan users, the mean (SD) sumatriptan dose equivalent difference between pre- and post-index was -26 (547) mg per month which represents a 2.9% decrease from the pre-index average dose equivalent (mean 911 mg per month). Among continuing opioid users, the mean (SD) morphine dose equivalent difference between pre-/post-index was 61 (997) mg per month which represents a 5.1% increase from the pre-index average dose equivalent (mean 1,191 mg per month). Among continuing ergot users, the mean (SD) ergotamine dose equivalent difference between pre-/post-index was 1.0 (6.8) mg per month which represents an 8.8% increase from the pre-index average dose equivalent (mean 11.4 mg per month).

**Table 5:** Dose equivalent changes in traditional acute migraine medications among continuing users during the 6-month post-index period, overall and by prior mAb use

	**Overall** ***N* = 78,574**	**Patients with prior mAb use**^**a**^ ***N* = 26,656**	**Patients without prior mAb use**^**a**^ ***n* = 51,918**
**Dose equivalent changes for triptans**			
Pre-index triptan use, n (%)	36,054 (45.9)	14,182 (53.2)	21,872 (42.1)
Patients with pre-index AND post-index triptan use, n (%)	20,677 (57.4)	9,081 (64.0)	11,596 (53.0)
Patients with continuing use of triptans^b^, n (%)	14,012 (67.8)	6,395 (70.4)	7,617 (65.7)
Pre-index dose per month, sumatriptan mg equivalents^c^, among patients with continuing use of triptans, mean (SD) [median]	911 (891) [750]	993 (930) [800]	843 (851) [650]
Post-index dose per month, sumatriptan mg equivalents^c^, among patients with continuing use of triptans, mean (SD) [median]	885 (854) [700]	951 (889) [750]	830 (820) [600]
Difference^d^ sumatriptan mg equivalents per month,^c^ mean (SD)	-26 (547)	-42 (563)	-13 (534)
Relative difference^e^	-2.9%	-4.2%	-1.5%
**Dose equivalent changes for opioids**			
Pre-index opioid use, n (%)	29,692 (37.8)	11,116 (41.7)	18,576 (35.8)
Patients with pre-index AND post-index opioid use, n (%)	20,745 (69.9)	8,121 (73.1)	12,624 (68.0)
Patients with continuing use of opioids^b^, n (%)	16,135 (77.8)	6,434 (79.2)	9,701 (76.8)
Pre-index dose per month, morphine mg equivalents^c^, among patients with continuing use of opioids, mean (SD) [median]	1,191 (2,586) [450]	1,124 (2,315) [450]	1,236 (2,751) [455]
Post-index dose per month, morphine mg equivalents^c^, among patients with continuing use of opioids, mean (SD) [median]	1,252 (2,601) [515]	1,165 (2,263) [500]	1,310 (2,801) [525]
Difference^d^ morphine mg equivalents per month,^c^ mean (SD)	61 (997)	41 (835)	74 (1,091)
Relative difference^e^	5.1%	3.6%	6.0%
**Dose equivalent changes for ergots**			
Pre-index ergot use, n (%)	1,255 (1.6)	718 (2.7)	537 (1.0)
Patients with pre-index AND post-index ergots, n (%)	501 (39.9)	300 (41.8)	201 (37.4)
Patients with continuing use of ergots^b^, n (%)	278 (55.5)	162 (54.0)	116 (57.7)
Pre-index dose per month, ergotamine mg equivalents^c^, among patients with continuing use of ergots, mean (SD) [median]	11.4 (12.5) [8.0]	11.1 (10.6)	11.8 (14.7) [8.0]
Post-index dose per month, ergotamine mg equivalents^c^, among patients with continuing use of ergots, mean (SD) [median]	12.4 (12.9) [10.0]	12.6 (10.4) [10.0]	12.2 (15.8) [8.0]
Difference^d^ ergotamine mg equivalents per month,^c^ mean (SD)	1.0 (6.8)	1.4 (6.3)	0.4 (7.3)
Relative difference^e^	8.8%	12.8%	3.7%

*mAb* monoclonal antibody, *SD* standard deviation

^a^Prior mAb (erenumab, galcanezumab, fremanezumab) use was evaluated during the 6-month period prior to the index date

^b^Continuing users were patients with use of the acute medications in the pre-index and who did not discontinue acute medication in the post-index period. Discontinuation of acute migraine medication was defined as no claims for the same therapeutic class of acute treatment during the 60 days prior to the end of the post-index period

^c^Dose in mg equivalents per month was calculated as the sum of quantity multiplied by the dose equivalent strength for claims during the 6-month period divided by 6 months

^d^Difference was calculated as the post-index value minus the pre-index value. Negative values represent a reduction in the post-index period while positive values represent an increase in the post-index period

^e^Relative change was calculated as (post-index mean dose—pre-index mean dose)/pre-index mean dose *100%

Although there were variations in dose equivalent changes for patients with prior mAb use and patients without prior mAb use, changes moved in the same direction for both subgroups. Triptan dose equivalents for continuing users were reduced for patients with prior mAb use (*n* = 6,395) and without prior mAb use (*n* = 7,617); -4.2% and -1.5%, respectively. Opioid dose equivalents for continuing users increased for patients with prior mAb use (*n* = 6,434) and without prior mAb use (*n* = 9,701); 3.6% and 6.0%, respectively. Ergotamine dose equivalents for continuing users also increased for patients with prior mAb use (*n* = 162) and without prior mAb use (*n* = 116); 12.8% and 3.7%, respectively.

## Discussion

Results from this study provide insight into real-world treatment patterns for patients initiating nAMSMs in the early period of their market availability in the U.S. A recent retrospective study conducted by Varnado et al. [20] used two commercial claims databases to evaluate treatment patterns in patients on mAbs and nAMSMs. In both databases, triptans (MarketScan: 64.8%; Optum: 60.3%) and NSAIDs (MarketScan: 37.6%; Optum: 39.9%) were the most commonly prescribed acute medication classes, and antiepileptics (MarketScan: 34.2%; Optum: 33.5%) and beta-blockers (MarketScan: 22.4%; Optum: 24.7%) were the most commonly prescribed preventive medication classes in patients with migraine. Differences between our results and the Varnado study may be due to differences in study design. While opioids and antidepressants were among the most commonly used traditional migraine medications in our study for acute and preventive use, respectively, Varnado et al. did not evaluate these medication classes. Varnado et al. required an overlap in anti-CGRP pathway mAb and nAMSM prescribing whereas we simply explored the use of nAMSM in the context of prior use of anti-CGRP pathway mAbs. The population in the Varnado et al. study had substantially more patients with comorbid anxiety, depression and hypertension. Additionally, the two study populations had important differences in insurance coverage. Specifically, our analysis included discount/coupon and cash coverage options, both not reported in Varnado et al. [20]. Despite these differences, treatment patterns for traditional anti-migraine medications during the pre-index period from Varnado et al. generally aligned with results in our study.

In our study, most patients initiating nAMSMs had prior treatment with traditional acute and preventive anti-migraine medications. One-third of patients had prior treatment with anti-CGRP pathway mAbs. A notable proportion of patients discontinued traditional acute and preventive anti-migraine medications after starting nAMSMs. Although changes in traditional acute anti-migraine medications were not evaluated in the context of changes in preventive medications or nAMSMs, the discontinuation of these medications may indicate that patients are substituting them with nAMSMs. As observed in a recent evaluation, this discontinuation could also be attributed to the initiation or ongoing use of mAbs, as patients may no longer need traditional acute anti-migraine medications while on effective preventive therapy [21]. We do note in our results that, among patients that remained on traditional acute anti-migraine medications, dose changes for these medications were minimal (i.e., < 10% overall), which may be due to chance variations in dose rather than a systematic change in prescribing patterns.

According to data available from prescription claims in our analysis, patients with prior use of mAbs had similar demographics and clinical characteristics as patients without prior use of mAbs, but they had greater pre-index use of traditional acute and prophylactic anti-migraine medications and more frequent recording of a neurologist as the index prescriber. Differences in discontinuation of traditional acute and preventive anti-migraine medications for patients with vs. without prior mAb use may be attributed to higher migraine burden in patients with prior mAb use, indicated by higher pre-index traditional acute and preventive anti-migraine medication use. Higher migraine burden in patients with prior mAb use is also reflected in post-index nAMSM refill patterns. Compared to patients without prior mAb use, a higher proportion of those with prior mAb use refilled index nAMSM and had a higher number of nAMSM refills.

Following nAMSM initiation, most patients (82%) with prior use of mAbs continued using a mAb (i.e., had one or more prescriptions in the post-index period). Among patients without prior use of mAbs, a small proportion (15%) initiated a mAb. Although data on safety and effectiveness of concomitant anti-CGRP pathway mAbs and gepants use are limited [22–26], our observations suggest combination use of these therapies in the real-world is common.

Of note, use of discount/coupon programs in 21% of patients at index and the high frequency of payer conversion observed in this study of unadjudicated open-source claims suggest that nAMSMs claims may not be consistently visible in adjudicated insurance claims data such as health plan claims. Due to the nature of commercial claims databases, missing prescriptions is common across therapeutic areas. In adults with prescription for opioids, diuretics, antiplatelet medications, or anticoagulants, only 68% of subjects had the same number of prescriptions in both LRx and PharMetrics databases [27]. Another study using these two databases also found missing statin claims in 20% of patients [28]. With anti-CGRP pathway mAbs, a recent study found that 46% of initial erenumab claims, 35% of initial fremanezumab claims, and 18% of initial galcanezumab claims were billed through a payer type that is not visible (i.e., due to free trial programs, bridge programs, denial conversions, or cash payments) in insurance claims databases [11]. Although the current study captured nAMSM claims across all payer types, further studies are warranted to examine the extent that nAMSM claims may be missing from adjudicated insurance claims databases.

This study had several limitations related to the data source and the retrospective observational design. As with other studies conducted using administrative claims databases, the indication intended for medications may not be captured, limiting the ability to determine whether the treatments are being used to treat migraine, a non-migraine condition, or both. Traditional acute and preventive treatments included in this study are known to be used to treat migraine, but some also have other indications. Over-the-counter medications, such as NSAIDS, might be underestimated in our study as are medications that can also be obtained without prescription including drug samples provided by physicians. In addition, we did not include treatment combinations often used for acute migraine management. Interpretation of the findings rely on the assumption that filled prescriptions were actually taken by patients; however, consumption of therapy cannot be confirmed. Claims for treatments and services billed outside of the open-source claims system may not be captured. This study applied proxy rules for data visibility and stability to minimize the potential for missing data and excluded patients who did not meet these requirements, which may bias the sample towards a population with more stable access to and utilization of the health care system. There may be confounding factors that caused discontinuation of traditional acute and preventive anti-migraine medications, such as side effects, lack of efficacy, or cost. The reason for discontinuation is not available in pharmacy claims data. As patients were not randomized to treatments and with limitations in the granularity of claims-based data, patients with prior mAb use may have had different patient characteristics and prior migraine treatment use (beyond those captured in our study) than patients without prior mAb use.

Despite the limitations, this real-world study utilized a claims database that captures a large, diverse patient population and provides insight into recent real-world treatment patterns for more than 78,000 patients initiating nAMSMs, more than 26,000 of whom had prior use of mAbs. The heterogeneous patient population obtained from the open-source claims database included all payer types and a wide geographical distribution across the US, providing results that could be considered representative of the US population.

## Conclusions

In this study of a large US population of patients initiating nAMSMs, most patients had prior treatment with traditional acute and preventive anti-migraine medications, and nearly one-third of patients had prior treatment with anti-CGRP pathway mAbs. After starting nAMSMs, more than one-third of patients discontinued at least one traditional acute treatment. Similarly, one-third of patients discontinued at least one traditional preventive treatment. The majority of those with prior mAb use continued mAb use after initiating nAMSMs. Among patients without prior mAb use, around 15% of patients newly started a mAb. To better understand the use of evolving treatment options for migraine, future research may focus on the integration of these medications into real-world clinical management of migraine. Not covered in our analysis, assessment of the safety and effectiveness of combination treatments will also be critical.

### Supplementary Information

**Additional file 1: Supplement Table 1.** Index to post-index payer conversion.

## Data Availability

Not applicable. Study utilizes open-source US pharmacy (LRx) and medical (Dx) claims data.
