# Supplementary material for: Treatment patterns for patients initiating novel acute migraine specific medications (nAMSMs) in the context of monoclonal antibodies (mAbs) targeting the calcitonin gene-related peptide (CGRP) pathway
Source: J Headache Pain. 2023 Nov 9;24(1):153. doi: 10.1186/s10194-023-01678-y (PMC10634163; doi:10.1186/s10194-023-01678-y)
Supplement: Supplementary file 1 — Additional file 1: Supplement Table 1. Index to post-index payer conversion. [file 10194_2023_1678_MOESM1_ESM.docx]

**Supplement, Table 1. Index to Post-index Payer Conversion**

|  | **Index payer commercial (N=50,607)** | | | **Index payer cash (N=2,997)** | | | **Index payer discounts/coupons (N=16,118)** | | |
| --- | --- | --- | --- | --- | --- | --- | --- | --- | --- |
| **Patients with ≥1 conversion (n, %)** | 5,316 | 10.5% |  | 1,598 | 53.3% |  | 4,863 | 30.2% |  |
| **Commercial** |  |  |  | 1,360 | 85.1% |  | 4,582 | 94.2% |  |
| Time (days) to conversion (mean, SD, median) |  |  |  | 73.5 | 49.9 | 63.0 | 78.9 | 48.5 | 70.0 |
| **Cash** | 747 | 14.1% |  |  |  |  | 134 | 2.8% |  |
| Time (days) to conversion (mean, SD, median) | 72.9 | 44.0 | 61.0 |  |  |  | 71.8 | 44.9 | 61.5 |
| **Discounts/coupons** | 4,346 | 81.8% |  | 188 | 11.8% |  |  |  |  |
| Time (days) to conversion (mean, SD, median) | 70.5 | 42.3 | 61.0 | 48.2 | 40.5 | 38.0 |  |  |  |
| **Medicaid** | 28 | 0.5% |  | 4 | 0.3% |  | 7 | 0.1% |  |
| Time (days) to conversion (mean, SD, median) | 96.1 | 47.3 | 94.0 | 31.3 | 21.7 | 36.5 | 86.0 | 53.4 | 64.0 |
| **Medicare** | 195 | 3.7% |  | 46 | 2.9% |  | 140 | 2.9% |  |
| Time (days) to conversion (mean, SD, median) | 94.2 | 49.2 | 94.0 | 47.0 | 53.9 | 24.0 | 69.5 | 48.5 | 56.5 |
| Time (days) to conversion (mean, SD, median) |  |  |  |  |  |  |  |  |  |
| **Patients without any conversion (n, %)** | 45,291 | 89.5% |  | 1,399 | 46.7% |  | 11,255 | 69.8% |  |
